# Microbuckling Instability and the Second Yield during the Deformation of Semicrystalline Polyethylene

**DOI:** 10.3390/polym12102208

**Published:** 2020-09-26

**Authors:** Zbigniew Bartczak, Alina Vozniak

**Affiliations:** Centre of Molecular and Macromolecular Studies, Polish Academy of Sciences, Sienkiewicza 112, 90-363 Łódź, Poland; awozniak@cbmm.lodz.pl

**Keywords:** semicrystalline polymer, deformation mechanisms, deformation instabilities, lamellae buckling, kinks, polyethylene

## Abstract

Deformation instabilities, such as microbuckling or lamellar fragmentation due to slip localization, play a very important role in the deformation of semicrystalline polymers, although it still not well explored. Such instabilities often appear necessary to modify the deformation path and facilitate strain accommodation in an energy-minimizing manner. In this work, microbuckling instability was investigated using partially oriented, injection-molded (IM) samples of high-density polyethylene, deformed by a plane-strain compression. Deformed samples were probed by SEM, X-ray (small- and wide-angle X-ray scattering: SAXS, WAXS), and differential scanning calorimetry (DSC). It was found that microbuckling instability, followed quickly by the formation of lamellar kinks, occurred in high-density polyethylene (HDPE) at a true strain of about e = 0.3–0.4, mainly in those lamellar stacks which were initially oriented parallel to the compression direction. This phenomenon was observed with scanning electron microscopy, especially in the oriented skin layers of IM specimens, where a chevron morphology resulting from lamellae microbuckling/kinking was evidenced. Macroscopically, this instability manifested as the so-called “second macroscopic yield” in the form of a hump in the true stress–true strain curve. Microbuckling instability can have a profound effect on the subsequent stages of the deformation process, as well as the resulting structure. This is particularly important in deforming well-oriented lamellar structures—e.g., in drawing pre-oriented films of a semicrystalline polymer, a process commonly used in many technologies.

## 1. Introduction

Semicrystalline polymers demonstrate a high ability to deform plastically, which appears to be one of their most essential properties, and has therefore been extensively studied (see reviews—e.g., [1,2,3,4]). The deformability of semicrystalline polymers can largely be attributed to their unique morphology, which at a basic level consists of thin alternating crystalline and amorphous layers, tightly linked together by chains crossing the interface. At temperatures above the glass transition, the rubber-like amorphous layers appear very soft and compliant compared to the crystalline layers; nevertheless, both components must deform cooperatively to satisfy the material integrity condition [4]. The deformation of a semicrystalline polymer turns out to be a complex process, involving various mechanisms active at different stages. The crystalline lamellae are principally deformed by crystallographic mechanisms [1,2,3,4,5], such as crystallographic slip (which is the dominant mechanism), twinning, or martensitic transformation. The non-crystallographic mechanism—strain-induced “melting-recrystallization” transition—has also been suggested by some authors as a possible mechanism [3,6,7]. The deformation of crystalline lamellae is supported by several micromechanisms operating in the amorphous phase, such as interlamellar shear, lamella separation, and the rotation of the lamella stacks. The active mechanisms can be fine-tuned through the interactions between adjoining crystalline and amorphous layers, as these layers are tightly and firmly interconnected and therefore forced to deform cooperatively. These interactions can also bring up some deformation instabilities, which in turn can modify the deformation sequence and/or activate alternative new mechanisms. The main deformation instabilities observed during the deformation of semicrystalline polymers are related to cavitation [8], lamella microbuckling [9], and slip localization, which results in the severe fragmentation of lamellae [10,11]. These instabilities often lead to very profound changes in morphology—e.g., the transformation of the initial lamellar structure into a microfibrillar one, which is commonly observed during tensile deformation. The instabilities mentioned above are mainly related to the amorphous phase and its interactions with crystalline lamellae [12].

One of the deformation instabilities is the microbuckling instability, resulting in lamellae undulation [9,10,11,13,14]. It occurs in those lamellae that were initially oriented with planes perpendicular to the direction of extension (which is parallel to the load direction in tensile deformation mode or perpendicular to it when in compression). Prior to microbuckling, these lamellae were not able to deform according to common crystallographic mechanisms because the corresponding shear stresses were too low to activate any of these mechanisms. Microbuckling instability leads to undulation and then to the formation of bigger folds or angular kinks of lamellae. This mechanism, although it can only accommodate minor strain, turns out to be very important for further deformation, as it causes a significant reorientation of the lamellae in kinks. This, in turn, increases the resolved shear stress in potential slip planes and enables conventional mechanisms such as crystallographic slip to be activated there. Moreover, kinking causes a collapse of the initial stiff skeleton of lamellae, which is often locally transformed into a chevron morphology (a wavy lamellar structure with lamellar folds or kinks comprising straight limbs and sharp hinges). These phenomena open up a new pathway for relatively easy plastic deformation due to the crystallographic slip systems for a significant part of the material volume, similar to that already available to other lamellae already oriented favorably for a slip. This transition initiated by microbuckling instability can manifest in the true stress–true strain curve as a low and wide local maximum (hump) located not too far beyond the yield point [10,11,15], which is commonly called the second yield point.

Folded or kinked structures due to layer buckling were often observed in various layered materials, mainly in response to the compressive force acting along the layers, but in some cases (e.g., liquid crystals or polymers) also in response to a tensile force perpendicular to the layers. Buckling has proven to be a common mechanism for layer deformation, recognized in various layered structures with very different length scales, ranging from the molecular length in smectic liquid crystals to about one kilometer in some natural rock formations [12]. Buckling is strain-controlled and results in an undulation of layers at a critical strain. As the strain increases further, these undulations grow and transform into bigger folds or angular kinks that may eventually form a chevron morphology [16]. Such morphology, originating from microbuckling instability, was also observed at the nano- and micrometer scale in block copolymers with a well-oriented lamellar morphology [16,17,18,19,20,21,22,23] and in semicrystalline polymers, either unoriented [4,9,24,25,26,27,28,29,30,31] or oriented [13,24,25,29,32].

Krumova et al. [9] argued that microbuckling, resulting in a chevron-like morphology, is a common deformation mode that can operate not only in materials with a well-ordered layered structure (e.g., layered rocks, oriented block copolymers with a lamellar structure, or oriented semicrystalline polymers) but also in ordinary ones—i.e., generally unoriented or only slightly oriented semicrystalline polymers. In such materials, it can operate locally—in places where the lamella stacks are locally oriented perpendicular to the extension direction (i.e., perpendicular to the tensile direction or along the compression direction, respectively). Consequently, microbuckling instability in semicrystalline polymers should be considered as a micromechanism as important as other accepted mechanisms of polymer plasticity. It is expected to activate and supplement these mechanisms as needed.

The mechanism of microbuckling in semicrystalline polymers is similar to that in other layered systems. The earliest studies of layer buckling phenomena were carried out in structural geology. Based on experimental observations of natural folds in rocks and model experiments, several theories of rock folding have been developed, mainly based on the elastic approach (see, e.g., [33]). Similar considerations and model calculations were later performed for liquid crystals and oriented lamellar block copolymers or semicrystalline polymers [13,16,18,21,22]. It was found that a necessary condition for buckling to occur is a layered morphology, consisting of stacks of almost flat and parallel alternating hard and soft layers and a large difference in stiffness between these layers. Strong interlayer coupling is also required, especially if buckling is to occur during tensile deformation, as in the case in polymeric materials. The cause of buckling is then a strong coupling and the Poisson effect—in a stack of layers drawn perpendicular to layering, the soft component accommodates most of the imposed strain. Due to the strong coupling with the hard phase, the lateral contraction due to the Poisson effect is prevented. This exerts strong compressive stress in the transverse direction on the hard layers. These layers withstand such a lateral compression and tensile stress in the direction normal to layers until the buckling instability takes place to alleviate lateral compressive stress.

According to theoretical considerations, the buckling of layers and their subsequent folding (rounded or angular) results from the natural tendency of materials to deform in a way that minimizes energy—the buckling of hard layers appears less expensive in free energy than the dilatation of soft layers [34]. Read et al. [16] found, using linear stability analysis, that buckling occurs at a critical strain due to geometrical instability in the equations of the elasticity of a layered material, actually approximated by a homogeneous anisotropic material in this approach. The initial small sinusoidal instability evolves into a kink or chevron shape, with further deformation beyond the point of instability. Using finite element analysis, Read proved that microbuckling instability can occur even in the purely elastic regime. Of course, nonlinearities, such as the plastic deformation of hard layers, can be involved, which then accentuate the kink profile [16]. The formation of angular folds such as kinks or chevrons was found to be energetically beneficial compared to round folds, especially when the contribution of the hard phase increases [34].

The elastic theories predicted the dependence of the critical strain of buckling on the ratio of the thickness of the soft and hard layers and the ratio of their modulus of elasticity [13,16]. Finite element modeling of highly oriented block copolymer [16] demonstrated that the first sinusoidal undulations were produced when the modulus ratio exceeded 500. In semicrystalline polymers above T_g_, this ratio is expected to be much higher than a few hundred, which indicates their susceptibility to microbuckling. Experimental evidence confirmed that microbuckling is driven by a large difference in the stiffness of the soft (amorphous) and hard (crystalline) layers [10,11].

In recent works [10,11,15], we studied several instabilities of plastic deformation in unoriented semicrystalline polyethylene subjected to plane-strain compression. Based on the mechanical, wide-angle X-ray scattering (WAXS), and small-angle X-ray scattering (SAXS) results, we found microbuckling instability occurring in PE samples with various structures around the true strain of e_crit_ = 0.2–0.4 and manifesting macroscopically as a low and wide hump observed in the true stress–true strain curve beyond the yield point, which can be considered as the second yield point. The dependence of the critical strain on the layer thickness and their elastic properties was found, which is consistent with the theoretical predictions. The aim of the present study was to gain more knowledge of this instability and to obtain direct microscopic evidence of the microbuckling mechanism operating in an unoriented or oriented semicrystalline polymer, such as linear polyethylene.

## 2. Materials and Methods

### 2.1. Material and Sample Preparation

The material used in this study was linear high-density polyethylene (HDPE) provided by Basell (Rotterdam, The Netherlands), with the molecular weight of M_w_ = 170,000 and M_n_ = 28,900, the melt flow rate (MFR) = 0.2 g/10 min (2.16 kg, 190 °C), and the density of 0.962 g/cm^3^.

Samples in the form of rectangular bars of 80 × 10 × 4 mm^3^ were produced by injection molding (IM) using a small laboratory injection molding machine (Proma, Toruń, Poland) at 190 °C and the mold temperature of 40 °C. The injection rate was approx. 3 cm^3^/s. The polymer was injected into the mold through the gate located close to one end of the bar. A few samples were also obtained by compression molding (CM) at 190 °C, followed by fast cooling.

Specimens of the size suitable to compression experiments in the channel-die were prepared by milling the IM or CM bars. The cutter and the milled material were continuously cooled with liquid to prevent any unwanted modification of its structure upon machining. The specimens had the dimensions of 50 × 8 × 3.85 mm^3^ along the injection direction (i.d.), transverse direction (t.d.), and normal direction (n.d.) of the injection-molded bars. Smaller specimens (4 × 8 × 3.85 mm^3^) were also prepared for the selected experiments.

### 2.2. Compression Tests 

Plane-strain compression tests were performed using the loading frame of a universal tensile testing machine (Instron, Norwood, MA, USA, Model 5582) and a compression fixture of the type of channel-die (channel length of 50 mm, width of 3.85 mm, and depth of 60 mm) made of hardened steel and equipped with an LVDT displacement sensor (Linear Variable Differential Transformer), mounted close to the specimen for precise strain determination. The specimens of the size of 50 × 3.85 × 8 mm^3^ were deformed using that die. In the selected experiments (e.g., compression along the injection direction), a small channel-die fixture—with a channel 8 mm long, 6 mm deep, and 3.85 mm wide—and miniature specimens (8 × 4 × 3.85 mm) were also used. The carefully machined specimens fitted precisely the width and length of the channel. Specimen surfaces contacting the walls of the die and the plunger were lubricated to minimize friction. Other details are given in [11,35].

All the deformation experiments were performed at room temperature with the constant true strain rate of ė = 0.001 s^−1^ controlled by the Bluehill^®^ II software of the testing machine; the current speed of the crosshead was constantly adjusted to the actual specimen height in order to keep the true strain rate constant. The true strain (Hencky) was calculated from the reduction in the specimen height (i.e., the size along the loading direction) using the following equation:

(1) $$e=-\int_{h_{1}=h_{o}}^{h_{1}=h}\frac{dh_{1}}{h_{1}}=\ln\left(\frac{h_{o}}{h_{o}-\Delta h}\right)=\ln\lambda ,$$

where *h_o_* denotes the initial height of the specimen, Δ*h* is the measured displacement of the plunger, and *h = h_o_* − Δ*h* is actual specimen height, while *λ = CR = h_o_/h* is the compression ratio. As the area of the sample under load in a channel-die remains constant (equal to the cross-section of the plunger) the true stress can be calculated with the following simple formula:

σ = F/A,(2)

where F is the measured force and A is the surface area of the plunger.

The coordinate system used throughout this paper is related to the geometry of the channel-die: the loading direction (LD) is the direction of the compressive force applied to the plunger; the constraint direction (CD) is perpendicular to the sidewalls of the channel; and the flow direction (FD) is parallel to the channel length—i.e., directed towards its opening. In most of the experiments, the LD coincided with the transverse direction (t.d.), the FD with the injection direction (i.d.), and the CD with the normal direction (n.d.) of the injection-molded specimen.

### 2.3. Characterization

**SAXS**: The lamellar structure of samples was probed with 2-dimensional small-angle X-ray scattering (2D-SAXS). The 1.2 m-long vacuum camera was coupled to a low-divergence X-ray CuK_α_ micro source, operating at 50 kV and 1 mA (sealed-tube micro-source integrated with multilayer collimation optics, producing a highly collimated beam with a divergence of 0.8 × 0.8 mrad^2^; GeniX Cu-LD by Xenocs, Grenoble, France). The collimation optics was combined with 2 additional hybrid scatterless slits assemblies (Xenocs) placed between the multilayer optics and the sample stage. These slit assemblies were each distanced 1.2 m from the other, forming the well-collimated beam of the square cross-section of less than 1 mm^2^. The slits use appropriately cut single crystals to shape the beam, whereby the parasitic scattering due to the crystalline grain boundaries in conventional slits is significantly reduced. Therefore, a normally necessary third guard slit or pinhole before the sample was no longer needed. The scattering produced by the sample was recorded with the Pilatus 100 K solid-state area detector of the resolution of 172 × 172 μm^2^ and module size of 83.8 × 33.5 mm (Dectris, Baden-Daetwill, Switzerland). Other details are given in [10,11].

**WAXS:** The effect of plastic deformation on the distribution of crystallite orientation was probed with 2-dimensional wide-angle X-ray scattering (2D-WAXS) in the transmission mode using a flat camera connected to a sealed-tube source, operating at 50 kV and 40 mA (CuK_α_ radiation, *λ* = 0.154 nm; Malvern Panalytical, Almelo, the Netherlands). The 2D-WAXS patterns were collected with a Pilatus 100 K area detector (Dectris).

**SEM:** The specimens for microscopic observations were prepared by permanganic etching according to the procedure developed originally by Olley et al. [36]. Prior to etching, the plane of interest in the sample was exposed by cutting with the ultramicrotome (Tesla, Brno, Czech Rep.) equipped with a freshly prepared glass knife. Typically, the samples were etched for approx. 1 h at room temperature in the mixture containing 1 wt.% KMnO_4_, dissolved in a 1:1 vol./vol. mixture of concentrated sulfuric and phosphoric acids. The composition of the mixture as well as the etching time were adjusted to the specific sample. Details of the procedure are given in [31]. The etched samples were coated with a 20 nm-thick gold layer and then examined with a scanning electron microscope, JEOL JSM-6010 LA (JEOL, Tokyo, Japan).

**DSC**: Thermal analysis of PE samples was conducted using a differential scanning calorimeter, DSC Q20 (TA Instruments, New Castle, DE, USA). The melting thermograms were recorded during heating from 0 to 200 °C with a rate of 10 °C/min under nitrogen flow. The weight crystallinity *X_c_* was calculated with the equation:

(3) $$X_{c}=\frac{\Delta h_{f}}{\Delta h_{f100}},$$

where Δ*h_f_* is the heat of the melting of the sample determined from the DSC melting curve and Δ*h_f_*_100_ = 293 J/g is the heat of the melting of 100% crystalline PE [37].

## 3. Results and Discussion

**Figure 1:**
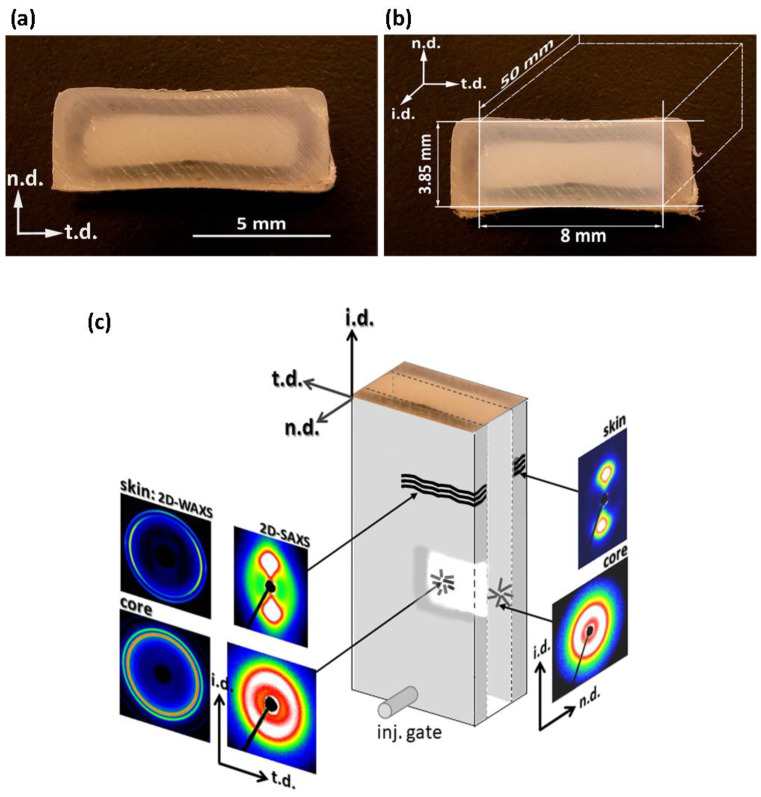
(**a**) The digital micrograph of the cross-section of the IM sample perpendicular to the injection direction (i.d.). On a black background, the opaque core layer looks light, and the transparent skin layer looks dark; (**b**) schematic drawing illustrating how specimens were cut from IM bars; (**c**) 2D-WAXS and 2D-SAXS images obtained in transmission, separately for the skin layer and the core layer, when the sample was illuminated along the normal direction (n.d.) or the transverse direction (t.d.).

Rectangular bar samples of HDPE were produced by injection molding (IM). Micrographs of the cross-section of the IM bar perpendicular to the injection direction revealed the core-skin morphology; an opaque core about 2 mm in thickness was surrounded by an almost transparent skin layer, about 1 mm thick—cf. Figure 1a. The IM bars demonstrated some shrinkage upon cooling, and therefore had to be carefully machined to obtain specimens with a regular parallelepiped shape that would fit very precisely into the channel of the channel-die compression fixture. The method is illustrated in Figure 1b. As a result, rectangular bars were prepared for deformation tests, with a cross-section of 8 × 3.85 mm^2^. The morphology of the bars obtained then consisted of a flat core layer, approx. 2 mm thick, sandwiched between two layers of skin, each approx. 1 mm thick; all these layers were normal to n.d. and extended full width along t.d., as shown schematically in Figure 1b,c. The contribution of the core and skin layers to the volume of the sample was approximately 50:50.

**Figure 2:**
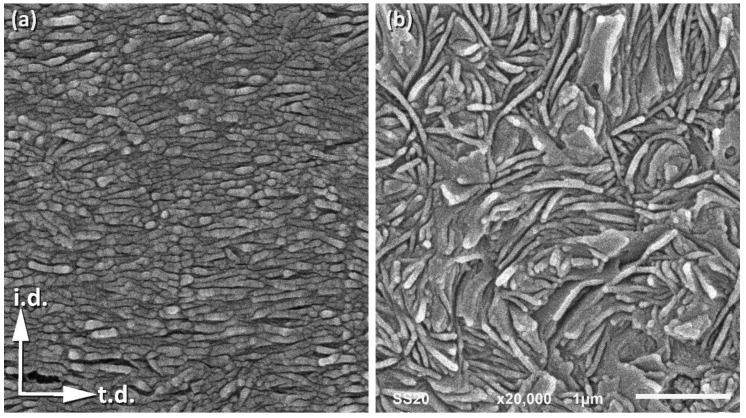
SEM micrographs of the skin (**a**) and the core (**b**) layers of the undeformed IM sample. The observation plane normal to n.d. was exposed by microtoming, followed by chemical (permanganic) etching to reveal details of the lamellar structure.

To examine the structure and morphology of the layers, small samples approximately 1 mm thick were taken from either the skin or the core layers of the IM bar and then examined with DSC, X-ray (both 2D-SAXS and 2D-WAXS), and scanning electron microscopy. The DSC melting data revealed that the skin layers contain crystals that melt at a slightly lower temperature than those in the core layer (134.7 vs. 135.4 °C), which implies that crystallites in the skin layers are only slightly thinner than in the core layer. The crystallinity of both layers is nearly the same: the values of X_c_ = 64.3 wt.% and X_c_ = 63.9 wt.% were estimated for the skin layer and the core layer, respectively. The results of X-ray scattering investigations are shown in Figure 1c. The SAXS images of the skin layer, when it was illuminated with X-ray along either n.d. or t.d., show a sharp two-point pattern that suggests a well-oriented lamellar structure with a lamella normal oriented preferentially along the injection direction, i.d. This finding is supported by 2D-WAXS images of the skin layer, where the observed position of the (110) and (200) reflections near t.d. can suggest the preferred orientation of the chain axis close to i.d.—i.e., similar to the preferred orientation of the lamella normal. The 2D-WAXS patterns of the skin layer (Figure 1) show relatively broad diffraction arcs of (110) plane centered along t.d., and (200) arcs also centered along t.d. Additionally, a second pair of (200) arcs was observed along i.d, although they were weaker than those observed along t.d. Such a diffraction pattern may suggest a crystalline texture consisting of two components. The main, stronger component consists of crystals preferentially oriented with poles of (110) and (200) planes perpendicular to i.d., which implies the chain axis close to i.d., just like the lamella normal. This is probably due to a structure formed under strong flow conditions, referred to as the “Keller/Machin II″ mode, where the lamellae are flat and the c-axis remains parallel to the injection direction [38,39]. The second, clearly weaker texture component, manifested by the broadening of the (110) arc and the diffraction arc of (200) centered along i.d., was probably produced by lamellae grown in the form of twisted ribbons, resulting in the rotation of the crystallographic a- and c-axis around the b-axis. This structure is termed the “Keller/Machin I″ mode, and is usually formed at weaker flow [39]—here, in the IM samples, possibly at the boundary of the skin layer just next to the core layer. In contrast to the skin layer, the core exhibited a uniform SAXS ring when illuminated along t.d., and an almost uniform scattering ring seen in the n.d.-view, with only a slightly higher scattering intensity along i.d. than along t.d. (cf. Figure 1c). At the same time, the corresponding 2D-WAXS images also show almost uniform diffraction rings, corresponding to the (110) and (200) planes of the orthorhombic unit cell of PE. All these features indicate an almost random orientation of the lamellae in the core layer, with only a trace of the preferred orientation perpendicular to i.d. The above-postulated preferred orientation of lamellae in the skin and core layers was confirmed by direct microscopical observations using a scanning electron microscope (SEM). Figure 2a,b present representative SEM micrographs of the skin and core layers, respectively. It can be easily seen in Figure 2a that virtually all the lamellae in the skin layer are flat and well-oriented perpendicular to i.d., while the micrograph in Figure 2b suggests no apparent preferred orientation of lamellae in the core layer. The lamellae in the skin layer seem to be relatively short compared to the core layer or fragmented, although it cannot be ruled out that this appearance of lamella in the micrograph is merely an artifact resulting from some overetching that may have occurred during sample preparation, particularly along the concentrations of dislocation lines or at sites of greater concentration of other defects that may give the impression of disrupted lamellae [31]. The lamellae in the core layer are long and appear to be organized in higher-order structures, such as spherulites (which can be recognized at a lower magnification), which are large enough to scatter visible light and thus make the core layer opaque. In contrast, the well-ordered skin layer (lacking higher-order morphological structures) does not scatter light so much, rendering it almost transparent.

**Figure 3:**
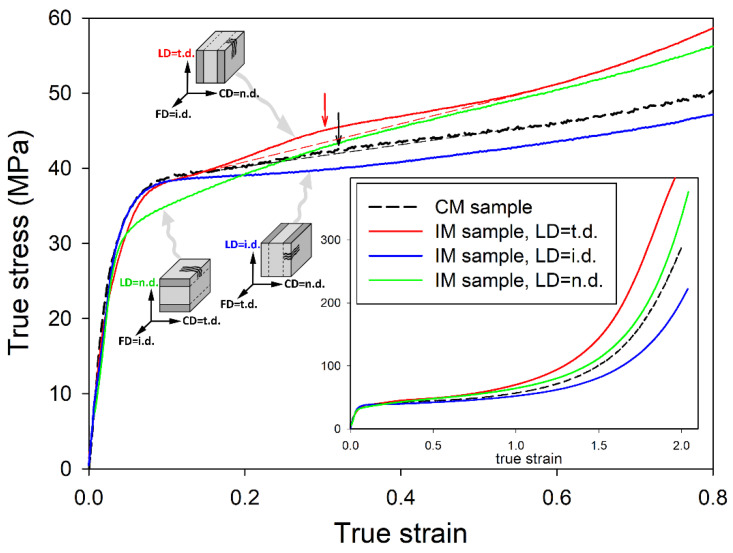
The true stress–true strain curves of the IM miniature samples deformed by plane-strain compression in the small channel-die. Different layer orientation with respect to the loading direction is shown for each curve. The main plot presents the enlarged initial part of the true stress–true strain curves, while the inset shows the same curves over the full range of stress and strain. Auxiliary dashed lines were drawn in the range of the second yield to better visualize the humps on the curves. The approximate position of the second yield point is indicated by an arrow.

The samples machined out from IM bars and having a well-defined three-layer sandwich morphology seemed very useful for investigating some aspects of the plastic deformation of a semicrystalline polymer, in particular the buckling instability [9,10,11,13,14,15]. Since both the skin and core layers are thick (approx. 1 and 2 mm, respectively) and separated by a thin transition layer, their deformation behavior can be considered almost independent of each other and can be assessed separately in a single compression experiment. The oriented (skin) and unoriented (core) components operate in parallel when the sample is loaded in any direction along the layering, while they act in series when it is loaded along the n.d., perpendicular to the layers. Figure 3 shows the mechanical response of IM sandwich-like samples when subjected to plane-strain compression, compared to a non-oriented, uniform CM sample (obtained by compression molding) deformed under the same conditions. The CM sample exhibited a spherulitic morphology similar to that observed in the core of the IM sample, with no trace of preferred lamellar orientation. The deformation of the CM sample and the underlying mechanisms were studied in detail and reported in our recent papers [10,11]. The main plot in Figure 3 shows the enlarged initial part of the true stress–true strain curves, while the inset shows the same curves over the full range of stress and strain. In two independent experiments, IM samples were loaded in two orthogonal directions parallel to the sample skin-core layering, either along the transverse direction or along the injection direction: LD = t.d. or LD = i.d., respectively. These correspond to the LD parallel or perpendicular to the plane of the preferred orientation of lamellae in the skin layer, respectively. Additionally, the sample compressed along the n.d. was also tested (LD was then perpendicular to the skin and core layers, as well as to the lamella normal in the skin layer).

Compared with the unoriented CM sample, IM samples compressed either along the i.d. or t.d. exhibited similar elastic behavior and yield stress, while the sample compressed along the n.d. demonstrated a slightly lower stress at yield. After passing the yield point, the IM (LD = i.d.) sample began to flow with a slowly increasing stress, which, however, remained all the time lower than that in CM. In contrast, the true stress observed in the IM (LD = t.d.) sample after passing the yield point continued to increase with the strain slightly faster than in CM and exceeded the stress observed in the CM sample at the true strain of e ≈ 0.2. The stress in IM (LD = t.d.) sample then remained higher than in the CM sample up to the highest strains. In the strain-hardening range, the slightly different slopes of the curves of the IM and CM samples ultimately led to a noticeable difference in the stress arising at the highest strains, approaching e = 2.0; the IM (LD = i.d.) (compressed perpendicularly to the preferred orientation plane of lamellae in the skin layers—i.e., along the preferred direction of the chain) demonstrated a lower strain hardening rate and consequently lower ultimate stress than CM, while the IM (LD = t.d.) sample—i.e., that compressed parallel to the preferred lamellar plane in the skin layer—exhibited a stronger strain-hardening effect than CM. The sample loaded along the n.d., (perpendicular to the skin core layering) demonstrated a stress–strain curve similar to that of the CM sample.

An important feature can be observed on the true stress–true strain curves shortly after passing the yield point, in the true strain range of about e = 0.3. It is a low and broad hump observed in the stress–strain curves of the CM, IM (LD = t.d.), and possibly IM (LD = n.d.) samples. Interestingly, such a hump cannot be discerned on the curve of the IM (LD = i.d.) sample. This hump is better developed and centered at the slightly smaller strain in the IM sample (LD = t.d.) than in the CM unoriented material—e ≈ 0.30 vs. 0.33. A very small hump is barely discernible on the curve of the IM (LD = n.d.) sample at a similar position to that seen in CM. A characteristic bulge similar to the hump discussed here was frequently observed in the stress–strain curves of various semicrystalline polymers shortly after passing the yield point, and was commonly referred to as the second yield [40,41,42,43]. This was often associated with the activation of a heterogeneous/coarse chain slip (block slip) [43,44,45,46,47] or with the activation of slip transverse to the chain direction [48]. In our recent works [10,11,15], we have postulated that the second yield point is rather associated with a certain deformation instability in the form of the microbuckling of lamellae followed by their kinking. This instability is experienced by stacks of lamellae, which at the beginning were specifically oriented parallel to the direction of compression (alternatively, in tensile deformation, by lamellae that were oriented perpendicular to the direction of drawing). Microbuckling, beginning as an elastic instability [16], leads to undulations of the lamellae that quickly turn into the formation of angular kinks, which involves irreversible plastic deformation and/or limited damage, especially in hinges of the sharp kink profiles [4,11,16,20,21]. The formation of such kinks is extremely important for the subsequent deformation behavior of the material, especially in some oriented structures, because kinking induces a quick and practically irreversible rotation of these lamellae, which were oriented initially parallel to the load and then underwent microbuckling, such as those in the polar region of spherulites or in oriented skin layers of the IM sample (when compressed perpendicularly to i.d.). Prior to buckling, these lamellae were not able to deform by any crystallographic mechanism due to too low resolved shear stress in the plane and direction of the potential slip system. The only deformation mechanism available for a stack of such lamellae was the so-called lamella separation, consisting of the dilatation of the interlamellar soft amorphous layer in the direction of their thickness, yet this mechanism was strongly constrained due to the very low compressibility of the rubber-like amorphous phase (a Poisson ratio close to *ν* = ½, a very high bulk modulus K), the large lateral sizes of the amorphous and crystalline layers, and the very strong coupling between layers; the interlamellar layer of such an almost incompressible amorphous material tends to contract laterally (in the plane of the layer) when stretched along its thickness to maintain a constant volume, however this contraction is severely restricted due to the large lateral dimensions of the layer compared to the thickness, and the very strong connectivity of the amorphous layer with the adjacent crystalline layers. These constraints are very effective in limiting lateral contraction and thus in “locking” this dilatational mode of deformation. This effect can also be explained in terms of high oedometric modulus, which can be expressed by:

(4) $$E_{oed}=\frac{E\left(1-\nu \right)}{\left(1+\nu \right)\left(1-2\nu \right)}\;=\;\frac{3K}{\left(1+v\right)}.$$

For a rubber-like amorphous polymer, *ν* is close to ½, hence *E_oed_* ≈ 2*K*—i.e., it appears very high, a few orders of magnitude higher than Young’s modulus *E*. A high value of the oedometric modulus *E_oed_* implies a very limited dilatation of the amorphous layers in response to tensile stress along the thickness direction—i.e., “locking” the lamella separation mechanism. As a consequence, the entire deformation process was temporarily locked locally with growing stresses, which would soon inevitably lead to deformation instability. To alleviate high lateral stress, the layered material may respond either with cavitation in the amorphous layers or with cooperative buckling of the crystalline/amorphous layers, possibly followed by their kinking and the formation of a chevron morphology. The particular response depends on the material properties and the stress field (tension/compression), but the microbuckling of layers often wins that competition, especially in the compression modes of deformation that usually suppress cavitation. The change in the orientation of lamellae, which occurred in the folds or kinks initiated by microbuckling instability, causes an increase in the resolved shear stress for the potential crystallographic slip system in these lamellae. This increase may appear to be sufficient to activate that slip mechanism. Moreover, kinking causes a collapse of the rigid lamellar structure, which may further relieve the lamellae of some of the previous morphological constraints. All these phenomena unlock the deformation process in these areas by opening up a new route for the easy plastic deformation of the crystalline lamellae—those reoriented rapidly in kink limbs can now deform relatively easily due to crystallographic slip, like other lamellae that had already been oriented favorably to such slip. This change allows the strain to be accommodated more easily and prevents excessive stress build-up, thus allowing further plastic deformation without the premature failure of the material.

It was established that the microbuckling is driven by significantly different levels of stiffness of the hard (crystalline) and soft (amorphous) layers and their strong interfacial connectivity through numerous tie-molecules and entangled loops [9,12,13,16]. It was found that it depends primarily on the ratio of stiffness of the layers and the concentration of the so-called stress transmitters at the crystal–amorphous interface (like tie-molecules, entangled loops, etc.) [10,11]. The stiffness of the layers, in turn, is determined by their thickness and the modulus of elasticity of the respective phase.

The microbuckling/kinking instability, which can facilitate the activation of new deformation mechanisms of lower plastic resistance, is often revealed in the stress–strain curve as a characteristic hump or bulge, which is commonly called the second macroscopic yield. We reported in our previous papers [10,11,15] a hump in the form of a very low and broad maximum related to the second yield in unoriented samples of several grades of polyethylene, which was observed around e ≈ 0.3–0.4. Concurrently, a rapid reduction in the scattering intensity in FD was noticed in the 2D-SAXS images in the same strain range. The scattering in FD is due to lamellar stacks that are oriented with the lamella planes parallel to LD (lamella normal parallel to FD). A sharp decrease in this component of scattering indicated the effective removal of such a specific orientation of lamellae with advancing strain. Moreover, the four-point pattern began to develop in the 2D-SAXS images (CD-view) shortly thereafter, at e ≥ 0.4, which revealed two emerging populations of lamellae, both inclined symmetrically to the direction of plastic flow and giving rise to chevron morphology. All these phenomena illustrated the same deformation mechanism, which in our opinion was the microbuckling instability that was quickly followed by the formation of lamellar kinks, activated at approx. e ≈ 0.3–0.4.

Similar observations were made for the samples studied in this work. First, we can note that an already reported hump in the stress–strain curves indicating the second macroscopic yield is larger in the IM (LD = t.d.) sample than in the CM sample, possibly because the population of lamella initially oriented parallel to LD in the IM (LD = t.d.) sample (including the skin layers where nearly all the lamellae were oriented in this way) is significantly larger than in the CM sample. The same large fraction of lamellae oriented parallel to LD can be found in the IM (LD = n.d.) sample, yet the core and skin layers here act in series upon load, as opposed to IM (LD = t.d.), where they operate in parallel. Consequently, the contribution of the oriented layers to the second yield in the IM (LD = t.d.) sample is greater than that in the IM (LD = n.d.) or CM. In contrast, the lamellae in the skin layer of the IM (LD = i.d.) sample are oriented perpendicular to LD, and thus the population of lamellae parallel to LD, which might contribute to microbuckling and kinking, is highly reduced compared to the IM (LD = t.d.) or even to the CM sample. Consequently, the IM (LD = i.d.) sample practically does not show a hump of the second yield in the true stress–true strain curve. 

**Figure 4:**
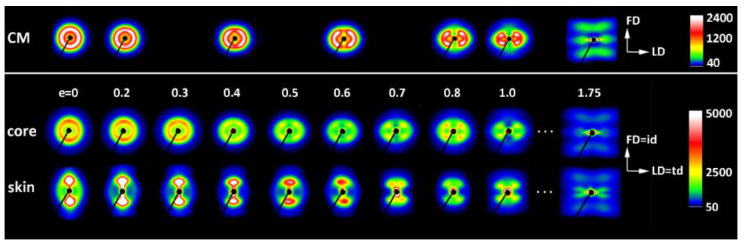
2D-SAXS patterns (CD-view) of the CM sample (upper row) and the core and skin part of the ID (LD = t.d.) injection-molded sample deformed by plane-strain compression along t.d. to the indicated true strain.

**Figure 5:**
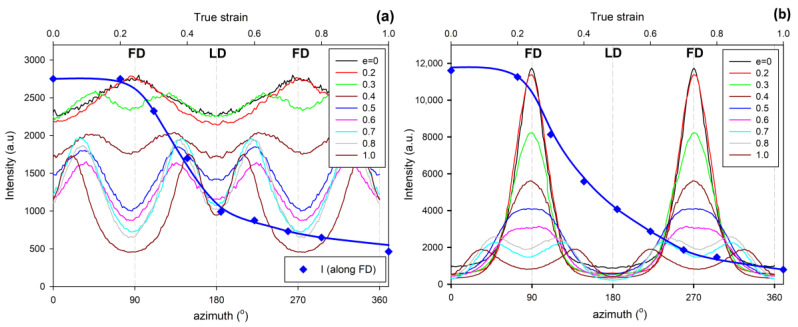
Azimuth scans in the range of scattering vector near the maximum of the SAXS images presented in Figure 4: (**a**) ID (LD = t.d.) core samples; (**b**) ID (LD = t.d.) skin samples. The thick blue line with diamond symbols shows the dependence of the intensity observed along FD (azimuth = 90° or 270°) on the strain applied (axis on the top).

**Figure 6:**
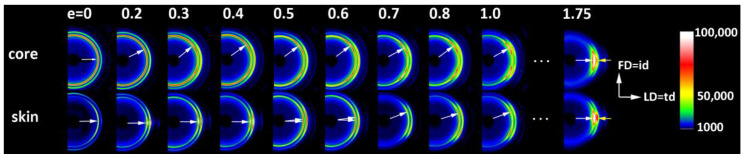
2D-WAXS patterns (CD-view) of the core and skin part of the ID (LD = t.d.) injection-molded sample deformed by plane-strain compression along t.d. to the indicated true strain.

In a separate experiment, IM samples were deformed to different, pre-selected strains. After the deformation to the desired strain, small specimens were cut from the skin and core layers of the deformed sample and examined with X-rays. Figure 4 shows a series of 2D-SAXS images in the CD-view (illumination of the sample with X-rays parallel to CD) of samples taken from the skin and the core layer of deformed IM samples (LD = t.d.). For comparison, images of CM samples deformed under analogous conditions are presented. In both series of images obtained for IM samples, one can observe an evolution of the X-ray pattern from an initial two-arc (core) or two-point (skin) pattern to the four-point pattern, which can be recognized at a strain of around e ≈ 0.4–0.5 and is fully developed above e = 0.7. Such a four-point pattern suggests the appearance of two populations of lamellae oriented symmetrically to the FD and contributing to the chevron morphology, which may be due to the lamella microbuckling and kinking. At e ≥ 1.0, the scattering images of the core and skin parts of the sample become very similar to each other and further change with the strain in a very similar fashion, indicating that the active deformation mechanism is identical for both the skin and the core parts of the sample. To illustrate this, the last row of Figure 4 shows SAXS images of the skin and core parts of the same sample deformed to e = 1.75, which are indeed very similar. The full sequence of the evolution of the SAXS pattern with increasing strain, including the e = 1.0–2.0 range not shown here, obtained for similar HDPE samples is presented in [10,11]. It has already been found that the main deformation mechanism at e ≥ 1.0 is an intense crystallographic slip in the direction parallel to the chain direction, which is already well advanced and therefore prone to a loss of stability and thus to localization. Such instability leads to lamellae fragmentation and then to a restructuring of the resultant blocks (a transformation known in tensile deformation as fibrillation), leading to high ordering and the formation of a new long period along the direction of plastic flow [10,11,49]. Figure 5 shows the azimuth profiles determined from the 2D images presented in Figure 4 in the range of the scattering vector around the value corresponding to the maximum intensity. These profiles demonstrate that the four-point pattern can be distinguished already at e = 0.3 in the core part of the sample—the maximum observed initially along FD splits into two separate peaks—and at e ≈ 0.4 in the skin layer (the maximum along FD broadens significantly and lowers; its shape clearly suggests two overlapping peaks). At the same time, a sharp drop in the scattering intensity measured along FD in the strain range of e ≈ 0.3–0.5 can be discerned, both in the core and skin parts of the sample, as illustrated in Figure 5. Figure 6 presents the 2D-WAXS patterns (CD-view) obtained for the same set of the deformed IM samples (LD = t.d.), already characterized by SAXS. It can be seen that the (110) and (200) planes, both parallel to the chain direction in the crystal lattice, are initially oriented preferentially close to LD (t.d.); the arcs of the high diffraction intensity of (110) and (200) planes can be found near the equator—marked with arrows in Figure 6—which suggests that the preferred orientation of the chain direction parallel to FD = i.d., as expected. As the strain increases, the maximum diffraction intensity of both planes (110) and (200) jumps to a new position, at some angle from t.d., which can be easily observed in the images of the sample core. This indicates the rotation of the chain axis away from the flow direction (FD = i.d.), which is consistent with the rapid rotation of lamellae detected by SAXS, all of which could have been expected for lamellae kinking. At higher strains, e > 1.0, the activity of the chain slip brings the strong orientation of the chain axis along FD (hence, the maxima of diffraction intensity of (110) and (200) planes are observed near LD—i.e., perpendicular to FD).

**Figure 7:**
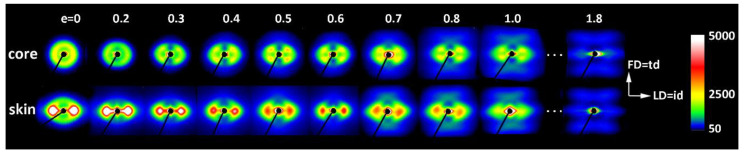
2D-SAXS patterns (CD-view) of the core and skin part of the ID (LD = i.d.) injection-molded sample deformed by plane-strain compression along the i.d. to the indicated true strain.

**Figure 8:**
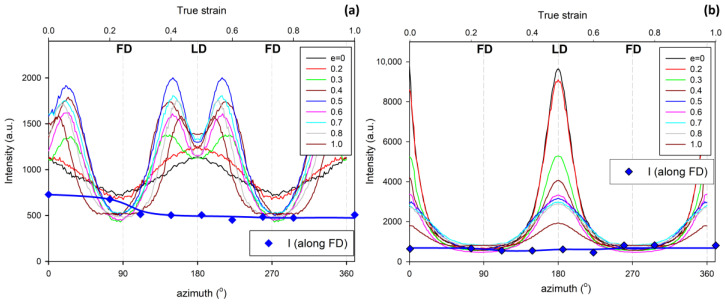
Azimuth scans in the range of scattering vector near the maximum of the SAXS images presented in Figure 5: (**a**) ID (LD = i.d.) core samples; (**b**) ID (LD = i.d.) skin samples. The thick blue line with diamond symbols shows the dependence of the intensity observed along the FD (azimuth = 90° or 270°) on the strain applied (axis on the top).

**Figure 9:**
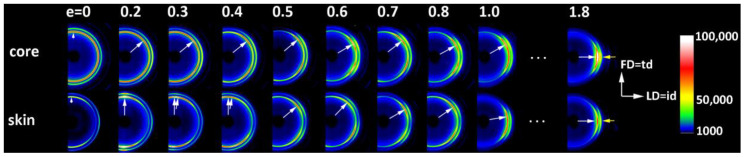
2D-WAXS patterns (CD-view) of the core and skin part of the ID (LD = t.d.) injection-molded sample deformed by plane-strain compression along the t.d. to the indicated true strain.

Figure 7, Figure 8 and Figure 9 show the X-ray dataset similar to that presented in Figure 4, Figure 5 and Figure 6, obtained for the core and skin specimens taken from IM samples compressed along i.d.: IM (LD = i.d.). Figure 7 presents 2D- SAXS images and Figure 9 2D-WAXS images, all obtained under X-ray illumination along CD (CD-view). Figure 8 shows the azimuth scans obtained from the SAXS images of Figure 7 (integrated within the range of the scattering vector around the value corresponding to the maximum intensity). It can be seen from Figure 7 and Figure 8 that the intensity of scattering along the FD, and therefore the population of the lamellae oriented perpendicular to FD (parallel to LD), is slightly reduced in the core part and is very small, almost absent, in the skin layer of the initial sample prior to deformation. Accordingly, the formation of the four-point pattern is observed upon deformation only in the core (e ≥ 0.3), while the skin shows practically no such signature in the SAXS pattern. There is only a reduction in height and a slight broadening of the peak centered at LD, which can be observed at e = 0.3–0.5. Instead of a four-point pattern, a new long period begins to emerge along FD at e ≈ 0.7, which can be read as a mark of extensive lamellae fragmentation and restructuring [10,11,49,50]. These observations are easy to understand, because for the LD = i.d. geometry, the relatively poorly oriented core layer does not differ significantly in lamellar orientation from the core of the IM (LD = t.d.) sample, as described earlier. Consequently, in the IM (LD = i.d.) sample, there are still enough lamellae oriented parallel to LD to respond to deformation with microbuckling and kinking, similarly to the case of samples with LD = t.d. On the other hand, the highly oriented skin layer of the IM (LD = i.d.) sample contains virtually no lamellae parallel to LD that are prone to microbuckling, which would lead to a four-point SAXS pattern. Most of the lamellae here are oriented perpendicular to LD, which makes them more vulnerable to cleavage and fragmentation under load. As a result, a new long period along the FD appears earlier in the SAXS pattern of the skin layer, instead of the four-point feature. The evolution of the crystal orientation revealed in the 2D-WAXS images presented in Figure 9 confirms that picture—there is a jump in the orientation of the chain direction (inferred from the rotation of (*hk*0) planes) in the core layer already at a low deformation, which is presumably related to the formation of lamellar kinks. On the other hand, the orientation of crystallites in the skin layer begins to change slowly at low strains and then faster at e ≈ 0.5, which is probably related to the progression of crystallographic slip and lamella fragmentation phenomena. These mechanisms ultimately led to the final well-oriented crystalline texture observed at high strain above e = 1.75 in both the skin and core layers.

**Figure 10:**
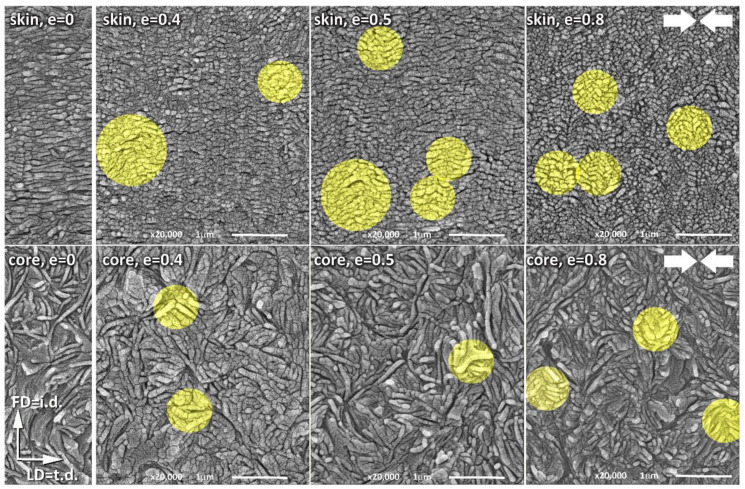
SEM micrographs of the LD-FD plane of the IM samples deformed to the indicated true strains of 0, 0.4, 0.5, and 0.8 by the plane-strain compression parallel to the t.d. direction. The direction of compression is indicated by thick arrows on the right-hand side. Upper row: samples taken from the skin layer. Bottom row: samples taken from the core layer. Samples were etched with permanganic etchant before observations to reveal the lamellar morphology. Bright yellow circles highlight the lamellar kinks that were formed during deformation.

As discussed above, the mechanical and X-ray scattering data, presented in our previous papers [10,11,15] as well as in this article, strongly support the hypothesis of deformation instability in the form of the microbuckling of lamellae, followed by their kinking and the local formation of a chevron morphology. The microbuckling instability leading to sinusoidal undulations of the lamellae is initially elastic [16], but the subsequent formation of kinks entails some plastic deformation and possibly damage phenomena around the hinge of the kink [11,21]. This manifests itself macroscopically in the stress–strain curve in the form of a hump, commonly referred to as the second macroscopic yield, as the microbuckling helps to overcome the temporary barrier to further deformation and allows the relatively easy plastic deformation to continue, facilitating new deformation mechanisms in the just-kinked lamellae—such as crystallographic slips, previously inaccessible to these lamellae before buckling. However, to obtain the direct evidence of such postulated behavior of the material during its deformation, microscopic observations of deformed samples that could confirm conclusions drawn from the X-ray data seemed highly desirable. Therefore, the microscopic observations of samples deformed to various strains were carried out using a scanning electron microscope (SEM). Both the skin and core of the sample were examined in the CD-view (LD-FD observation plane). The LD-FD plane of observation was first exposed by microtoming, and then the lamellar morphology in this plane was revealed by permanganic etching, which can selectively etch the amorphous material of the interlamellar layers [36]. Figure 10 presents the SEM micrographs of the skin and core layers of the IM samples compressed along the t.d. to a true strain ranging from 0 to 0.8. The narrow micrographs presented in the first left column illustrate the initial morphology of the sample before deformation. These micrographs show the same morphology as already observed in Figure 2, just to recall the essential features of the morphology of the skin (upper row) and core (bottom row) layers. As was already discussed, the lamellae in the skin layer are well-oriented parallel to the t.d., while the preferred orientation of lamellae in the core layer is almost invisible. These lamellae in the core layer seem to be organized in higher-order structures, such as spherulites, which can be recognized at a lower magnification. The lamellae in the skin layer appear shorter than those in the core layer, although this look can be artificial, as has already been discussed earlier in this section. The micrographs of the sample deformed to a relatively low true strain of e = 0.4, which is within the strain range of a hump indicative of the second yield in the stress–strain curve, show some changes in the orientation of lamellae due to the active deformation mechanisms. In some areas (marked with highlighted circles on the micrographs), an early stage of the formation of lamellar kinks can be observed. These early kinks, which are not yet well developed, can be recognized in both the skin- and core structures. As illustrated by the micrographs of samples compressed to the true strain of e = 0.5 and e = 0.8, presented in the middle and on the right-hand side of Figure 10, the kinks become more mature and sharper as the strain increases. Moreover, the number of kinks that can be identified in each micrograph increases with the increasing strain, especially in the skin layer, which exhibits a well-oriented lamellar structure. This finding is reasonable because the number of lamellar stacks parallel to the direction of the load, which are the most susceptible to microbuckling and kinking, is significantly greater in a well-oriented skin layer than in the core layer, showing only a poor orientation. At e = 0.8, fairly well-developed and regular kinks can be easily seen in the skin layer, which locally lead to a chevron morphology. Now, virtually all the lamellae change their orientation from the initial, parallel to LD = t.d., to the inclined one. Lamellae inclined with respect to the LD experience a significantly higher resolved shear stress in potential slip systems than they do in the initial orientation parallel to LD, therefore crystallographic slip mechanisms can be easily activated in such lamellae, reoriented in kinks, to continue the deformation process [4,49,51]. The same phenomena also take place in the much less oriented core part of the sample, although here the kinks, which are much less numerous than in the oriented skin layer, can only locally support the main deformation mechanism—the crystallographic chain slip, which was activated earlier, at the yield point, in other lamellae already oriented favorably for the slip mechanism.

We believe that the presented micrographs, clearly showing the formation of lamellar kinks already at relatively low strains around e ≈ 0.4, provide convincing evidence of the microbuckling deformation instability, which in our opinion is the mechanism responsible for the second macroscopic yield that is frequently observed in semicrystalline polymers.

## 4. Conclusions

The study of the deformation of HDPE allowed concluding that the deformation process of a semicrystalline polymer depends not only on the common deformation mechanisms that are active in a broad range of strain, such as the crystallographic slip or interlamellar shear in amorphous layers. As already established, these basic mechanisms are occasionally supported by other specific mechanisms, such as twinning or martensitic transformation, when possible [1,2,4]. Furthermore, cavitation—the instability affecting primarily the amorphous phase—appeared important for the deformation of also the crystalline phase in certain deformation modes, such as uniaxial extension, and for the final structure and morphology of the deformed material. Our recent studies revealed, in turn, that some more subtle phenomena, like other deformation instabilities—e.g., microbuckling or lamellae fragmentation due to slip localization—may also play a very important role in the deformation sequence of a semicrystalline polymer [10,11,15]. These instabilities are often needed to modify the deformation sequence or enable the activation of new mechanisms in order to facilitate strain accommodation in a way that minimizes energy consumption and prevents the premature failure of the material.

In this work, the important role of the deformation instability through lamellae microbuckling was studied in linear polyethylene (HDPE), selected as a representative semicrystalline polymer. The microbuckling instability occurs early in HDPE at a relatively low true strain of around e = 0.3. It is experienced by lamellar stacks, which are specifically oriented parallel to the compression direction (actually, the same can happen in tensile deformation when the lamella stacks are drawn perpendicularly to layering). Microbuckling, initially elastic, quickly transforms with increasing strain into the cooperative folding of lamellae and the formation of angular kinks, involving non-reversible plastic deformation and/or limited damage, especially in the hinges of sharp kink profiles. Microbuckling, resulting in the formation of lamellar kinks and sometimes a chevron-like morphology, as evidenced by the X-ray and electron microscopic observations reported in this article, appears extremely important for the subsequent deformation behavior of a semicrystalline polymer, especially in some oriented structures, since kinking induces the rapid and practically irreversible rotation of these lamellae, which were oriented initially parallel to the compression (such as, e.g., those in polar regions of spherulites or in oriented skin layers of the injection-molded sample when compressed perpendicularly to the injection direction). Before buckling, these lamellae were not able to deform by any crystallographic mechanism due to the too low resolved shear stress in the plane and direction of potential slip; consequently, the deformation was temporarily locked locally in these areas. The reorientation of the lamellae in the kinks initiated by microbuckling results in an increase in the resolved shear stress for potential crystallographic slip systems. At a certain strain, this increase may appear sufficient to activate the slip mechanism and initiate then an extensive plastic deformation. This opens up a new deformation route of relatively easy plastic deformation—the kinked lamellae can now deform by crystallographic slip, like other lamellae that have already been oriented favorably for a crystallographic slip. This provides easier strain accommodation and precludes excessive stress build-up, thus preventing the premature failure of the material. This transition induced by microbuckling/kinking instability frequently manifests itself in the stress–strain curve as a broad hump, referred to as the second macroscopic yield.

Microbuckling/kinking instability, although appearing to be a common deformation mode that may operate in the deformation of materials of various structures and morphologies, can be particularly important in deforming well-oriented lamellar structures—e.g., in the drawing of the pre-oriented films of semicrystalline polymer, a process commonly used in many technologies, such as the production of porous membranes.
